# Pair‐Feeding Study Designs Can Create Biases and Inflate Type I Error Rates: A Simulation Study

**DOI:** 10.1002/oby.70079

**Published:** 2025-11-24

**Authors:** Wasiuddin Najam, Daniel E. Kpormegbey, Deependra K. Thapa, Bret Rust, Lilian Golzarri‐Arroyo, Lance H. Baumgard, Louis Wai‐Tong Fan, Joseph G. Ibrahim, Edith J. Mayorga, David B. Allison

**Affiliations:** ^1^ Department of Epidemiology and Biostatistics, School of Public Health‐Bloomington Indiana University Bloomington Indiana USA; ^2^ Department of Applied Health Science, School of Public Health‐Bloomington Indiana University Bloomington Indiana USA; ^3^ Biostatistics Consulting Center, School of Public Health‐Bloomington Indiana University Bloomington Indiana USA; ^4^ Department of Animal Science Iowa State University Ames Iowa USA; ^5^ School of Data Science and Society University of North Carolina Chapel Hill North Carolina USA; ^6^ Department of Organismic and Evolutionary Biology Harvard University Cambridge Massachusetts USA; ^7^ Department of Biostatistics University of North Carolina Chapel Hill North Carolina USA; ^8^ Children's Nutrition Research Center, USDA‐ARS Baylor College of Medicine Houston Texas USA

**Keywords:** bias, data analysis, pair‐feeding, rigor, statistical science, study design, type one error

## Abstract

**Objective:**

Pair‐feeding is a study design element where one group's food intake is provided to another group to assess whether a treatment effect is independent of food intake. Investigators often assume equivalent food intake across experimental conditions and exclude it from the statistical analysis. However, the impact of this practice on type I error (T1Er) rates has not been quantified.

**Methods:**

We conducted a Monte Carlo simulation in which animals were assigned baseline weights and food intakes, then randomized to non‐pair‐fed or pair‐fed groups. Daily food intake for both groups was initially drawn from the baseline food intake distribution. For pair‐fed animals, food intake was truncated if it exceeded the previous day's intake of the matched non‐pair‐fed animal (individual pair‐feeding) or the group's average (group pair‐feeding). Weight changes were calculated as a function of food intake, and final weight change was analyzed with and without adjusting for mean food intake.

**Results:**

Both individual and group pair‐feeding inflated T1Er rates ranging from 0.12 to 0.71 in unadjusted models. However, adjustment for food intake reduced error rates to around 0.05.

**Conclusions:**

Under some circumstances, pair‐feeding designs can inflate T1Er rates. Investigators can mitigate this inflation by adjusting the analyses for food intake.

**Study Importance**

- What is already known?
  ○ Pair‐feeding is used to control for food intake in animal studies in an attempt to equalize food intake across groups. In studies using pair‐feeding, food intake is usually not controlled for in the statistical analysis.
  ○ Common pair‐feeding designs include individual and group pair‐feeding.
- What does this study add?
  ○ Both individual and group pair‐feeding can inflate type I error rates when food intake is not adjusted for in the statistical analysis.
  ○ Adjustment for food intake in the analysis can reduce type I error rates to nominal alpha levels.
- How might these results change the direction of research?
  ○ Our results highlight the need to include food intake as a covariate in the statistical analysis of pair‐feeding studies.
  ○ Our results may prompt a reevaluation of previously published findings where food intake was not included in the statistical analysis of pair‐feeding studies.

## 1 Introduction

The amount of food consumed by an individual influences their physiological and biochemical processes, which in turn can influence the effect of an intervention on an outcome of interest in many areas of research, particularly in experimental studies involving nutrition, drugs, and metabolism [1, 2, 3, 4, 5, 6]. In some of these experimental studies, the main aim is to examine the effect on an outcome of interest caused solely by the intervention, independent of any effect on food intake (i.e., with food intake eliminated as a mediator or post‐randomization confounder, recognizing that the distinction between a mediator and a post‐randomization confounder depends on the investigator's conception of the estimand [7]). To achieve this goal, it is important to isolate the effect of the intervention from the effect of differences in food intake on the outcome of interest. A common study design element used to address this issue is pair‐feeding.

Pair‐feeding is an experimental study design element in which the amount of food consumed by one group (non‐pair‐fed group) is provided to another group (pair‐fed group) to eliminate group differences in the total amount of food consumed per animal [3, 6, 8]. Pair‐feeding is usually used with nonhuman animals but has even been proposed for use in human studies [9]. The intent of pair‐feeding is to create circumstances such that any observed differences in the outcome of interest between groups are not influenced by differences in food intake and can be attributed to the intervention. Pair‐feeding studies are commonly approached in two ways: group pair‐feeding and individual pair‐feeding. In group pair‐feeding, the mean food intake consumed by the non‐pair‐fed group in a day is provided to every animal in the pair‐fed group [10, 11], usually on the subsequent day. Therefore, the maximum amount of food consumed in the pair‐fed group by an animal is “capped” at the mean food intake of the non‐pair‐fed group on the previous day. By contrast, in individual pair‐feeding, individual animals in pair‐fed and non‐pair‐fed groups are matched based on some covariates such as body weight, and the food consumed by an animal in the non‐pair‐fed group is provided to its matched or paired animal in the pair‐fed group [12]. As a result, a pair‐fed group animal's food intake may be lower than its non‐pair‐fed matched animal's food intake on the previous day, but it cannot exceed the food consumed by its matched animal. The decision of whether to designate the treatment or the control group as the pair‐fed or non‐pair‐fed group depends on whether the intervention increases or decreases food intake. If the intervention is expected to increase food intake, the control group is designated as the non‐pair‐fed group with ad libitum food access, while the treatment group is the pair‐fed group [13]. In contrast, if the intervention is expected to decrease food intake, the treatment and control groups will be designated as non‐pair‐fed and pair‐fed groups, respectively [14].

The stable unit treatment value assumption (SUTVA) is an a priori assumption in causal inference that includes the consistency or treatment variation irrelevance assumption and no interference assumption [15, 16]. The no interference assumption states that the outcome value for an individual is not dependent on the intervention received by other individual(s) [16]. In the pair‐feeding study design, the feeding behavior (i.e., the amount or timing of food intake) of the non‐pair‐fed group directly determines the feeding behavior of the pair‐fed subject or group, which can influence the outcome of interest in the pair‐fed group. This violates the SUTVA.

The fundamental assumption in pair‐feeding studies is that the food intake of the control and treatment groups is equal in expected value. This assumption allows investigators to test the null hypothesis that there is no effect of treatment assignment on the outcome of interest, independent of food intake. As a result, most investigators do not account for food intake in their statistical analysis. However, because the SUTVA is violated in pair‐feeding designs, causal effect estimates in pair‐feeding designs that do not account for food intake might be biased. To our knowledge, no study has quantified this postulated bias introduced by pair‐feeding. To address this research gap, we investigated whether and how pair‐feeding designs create biases and inflate type I error rates.

## 2 Methods

### 2.1 Simulation Study

We used Monte Carlo simulations to evaluate type I error rates for both group and individual pair‐feeding study designs. The simulation study followed several steps, as detailed here.

#### 2.1.1 Baseline Characteristics and Treatment Assignment

Let j=1,2,…,m denote the number of animals per group, so there are 2j total animals (j in the control group and j in the treatment group). Baseline body weights $W_{j}\left(0\right)$ in grams and food intake $F_{j}\left(0\right)$ in kilocalories (kcal) for each animal are drawn independently from normal distributions. Because pair‐feeding studies have predominantly been performed in mice, for our simulation, we adopted the mean and variance of a normal distribution for mice based on a previously published study [17].

Wj0iid~Nμw=33σw=0.6

Fj0iid~Nμf=13.3σf=0.3

Animals are randomly assigned to the pair‐fed or non‐pair‐fed group in a 1:1 ratio, ensuring j animals per group.

#### 2.1.2 Pairing in Individual Pair‐Feeding Design

In the individual pair‐feeding design, animals in the pair‐fed and non‐pair‐fed groups are ranked by their baseline body weight in ascending order. The *j*th smallest ranked animal in the pair‐fed group, with baseline weight $w_{\left(\;j\right)}^{\;p}\left(0\right)$, is paired with the *j*th smallest ranked animal in the non‐pair‐fed group, with baseline weight $w_{\left(\;j\right)}^{\mathrm{np}}\left(0\right)$. Thus, in the individual pair‐feeding design, the pairing is defined as:

$$w_{\left(\;j\right)}^{p}\left(0\right)\;\text{is paired with}\;w_{\left(\;j\right)}^{\mathrm{np}}\left(0\right)$$

where the index $\left(\;j\right)$ denotes the animal's rank based on body weight, with p representing the pair‐fed group and np representing the non‐pair‐fed group.

#### 2.1.3 Food Intake for Non‐Pair‐Fed Group Throughout the Study

For each day i=1,2,…,n, the daily food intake (kcal) for non‐pair‐fed $F_{j}^{\mathrm{np}}\left(i\right)$ animals is generated independently from the same normal distribution as the baseline:

Fjnpiiid~Nμnp=13.3σnp=0.3,fori=1,2,3,….,nandj=1,2,…,m

#### 2.1.4 Food Intake for Pair‐Fed Group Throughout the Study

Initially, for each day i=1,2,…,n, the daily food intake (kcal) for pair‐fed $F_{j}^{p}\left(i\right)$ animals is generated independently from the same normal distribution as the baseline:

Fjpiiid~Nμp=13.3σ=0.3,fori=1,2,3,….,nandj=1,2,…,m

Subsequently, the food intake for every pair‐fed animal is truncated based on the pair‐feeding design, as follows:

- *Group Pair‐Feeding*: The food intake of a pair‐fed animal is truncated if it exceeds the previous day's mean food intake of the non‐pair‐fed group, defined as:
$${\bar{F}}^{\mathrm{np}}\;\left(i-1\right)=\frac{1}{m}\;\sum_{j=1}^{m}F_{j}^{\mathrm{np}}\left(i-1\right)$$

The truncated food intake for a pair‐fed animal is:

$$\begin{array}{c} F_{j}^{p}\left(i\right)=\left\{\begin{array}{c} F_{j}^{p}\left(i\right),\;\;\text{if}\;F_{j}^{p}\left(i\right)\leq {\bar{F}}^{\mathrm{np}}\left(i-1\right)\\ {\bar{F}}^{\mathrm{np}}\left(i-1\right),\;\text{if}\;F_{j}^{p}\left(i\right)>{\bar{F}}^{\mathrm{np}}\left(i-1\right) \end{array}\right. \end{array}$$

- *Individual Pair‐Feeding*: The food intake of a pair‐fed animal is truncated if it would otherwise exceed the previous day's food intake of its paired animal from the non‐pair‐fed group. For the *j*th ranked paired animal in the pair‐fed group, the truncated food intake is:
$$\begin{array}{c} F_{\left(\;j\right)}^{p}\left(i\right)=\left\{\begin{array}{c} F_{\left(\;j\right)}^{p}\left(i\right),\;\text{if}\;F_{\left(\;j\right)}^{p}\left(i\right)\leq F_{\left(\;j\right)}^{\mathrm{np}}\left(i-1\right)\\ F_{\left(\;j\right)}^{\mathrm{np}}\left(i-1\right),\;\text{if}\;F_{\left(\;j\right)}^{p}\left(i\right)>F_{\left(\;j\right)}^{\mathrm{np}}\left(i-1\right) \end{array}\right. \end{array}$$

where $F_{\left(\;j\right)}^{\mathrm{np}}\left(i-1\right)$ is the food intake of the *j*th ranked paired animal from the non‐pair‐fed group on day i−1.

#### 2.1.5 Weight Change Calculation

We define $F_{j}\left(0\right)$ as the baseline food intake for animal *j*, representing the amount necessary to maintain its body weight. To assess changes in body weight as a function of food intake, we compared the food consumed on day *i*, denoted as $F_{j}\left(i\right)$, with the baseline amount required for weight maintenance used to determine whether intake was lower or higher. If the animal consumed less or more than the maintenance amount, the animal would lose or gain weight, respectively. Therefore, the body weight of animal j on day i, denoted as $W_{j}\left(i\right)$, is updated recursively based on the previous day's weight and the difference in current day's food intake from the baseline maintenance level as follows:

$$W_{j}\left(i\right)=W_{j}\left(i-1\right)+\left(F_{j}\left(i\right)-F_{j}\left(0\right)\right)*\delta$$

where $\delta =\frac{1}{3500}$ represents the weight change per unit of calorie intake.

#### 2.1.6 Statistical Analysis

The study outcome was the total body weight change, calculated as the difference between each animal's weight at the end of the study $W_{j}\left(n\right)$ and its baseline weight $W_{j}\left(0\right):∆W_{j}=W_{j}\left(n\right)-W_{j}\left(0\right)$. In the group pair‐feeding design, two linear regression models were used to evaluate the type I error rate for the treatment assignment effect coefficient $\left(\beta_{1}\right)$ on the difference in weight change between the pair‐fed and non‐pair‐fed groups: one without any covariate and the other including the animal's average daily food intake over *n* days (within‐animal mean food intake) as a covariate. However, in the individual pair‐feeding design, four models were used to evaluate the type I error rate for the treatment assignment effect coefficient $\left(\beta_{1}\right)$ on the difference in weight change: two linear regression models and two linear mixed‐effects models. One linear regression model included no covariate, while the other included within‐animal mean food intake as a covariate. One linear mixed‐effects model included only pairing as a random effect to account for clusters created due to pairing, while the other included both pairing as a random effect and within‐animal mean food intake as a covariate (see Appendix S1).

To calculate type I error rates, we ran simulations with combinations of sample sizes $\left(2J=16\;\text{or}\;80\right)$ and study durations $\left(n=15\;\text{or}\;50\right)$ for *k* = 1, 2, …, 10,000 iterations each. The empirical type I error rate was computed as the proportion of simulations where the *p* value for the treatment effect coefficient was less than 0.05 under the null hypothesis of no true treatment effect:

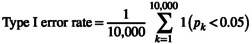

where $p_{k}$ is the *p* value for the treatment effect coefficient $\left(\beta_{1}\right)$ in the *k*th simulation, and 1(·) is the indicator function that is equal to 1 if $p_{k}<0.05$ and 0 otherwise. The chi‐square test was then used to compare the observed type I error rate with nominal type I error rate (alpha level) of 0.05.

Additionally, to determine whether the differences in food intake between pair‐fed and non‐pair‐fed groups are different in group versus individual pair‐feeding designs, we first calculated the mean of differences in overall food intake between the pair‐fed and non‐pair‐fed groups for each simulation. We then applied a *t*‐test to assess whether these differences varied significantly between the two pair‐feeding design types.

### 2.2 Reanalysis of an Existing Experimental Study

To illustrate the practical implications of pair‐feeding designs with and without food intake adjustment, we reanalyzed data from a randomized experimental group pair‐feeding study by Mayorga et al. [18]. This study examined the impact of dietary live yeast supplementation on growth performance and key metabolic and inflammatory biomarkers in pigs under heat stress and nutrient restriction. Pigs were randomly assigned to one of six groups: thermoneutral conditions with ad libitum control diet (TNCtl); thermoneutral conditions with ad libitum live yeast‐supplemented diet (TNYst); heat stress with ad libitum control diet (HSCtl); heat stress with ad libitum live yeast‐supplemented diet (HSYst); thermoneutral conditions pair‐fed to HSCtl control diet (PFCtl); and thermoneutral conditions pair‐fed to HSYst live yeast‐supplemented diet (PFYst). Average daily body weight gain (ADG) was one of the reported outcomes. We reproduced the original study's results for ADG and reanalyzed the data by adjusting for food intake, which was not accounted for in the original analysis. Additionally, we also included subject, pigs in this case, as a random effect in our reanalysis to better model individual variability.

## 3 Results

### 3.1 Simulation Study

#### 3.1.1 Group Pair‐Feeding

**FIGURE 1:**
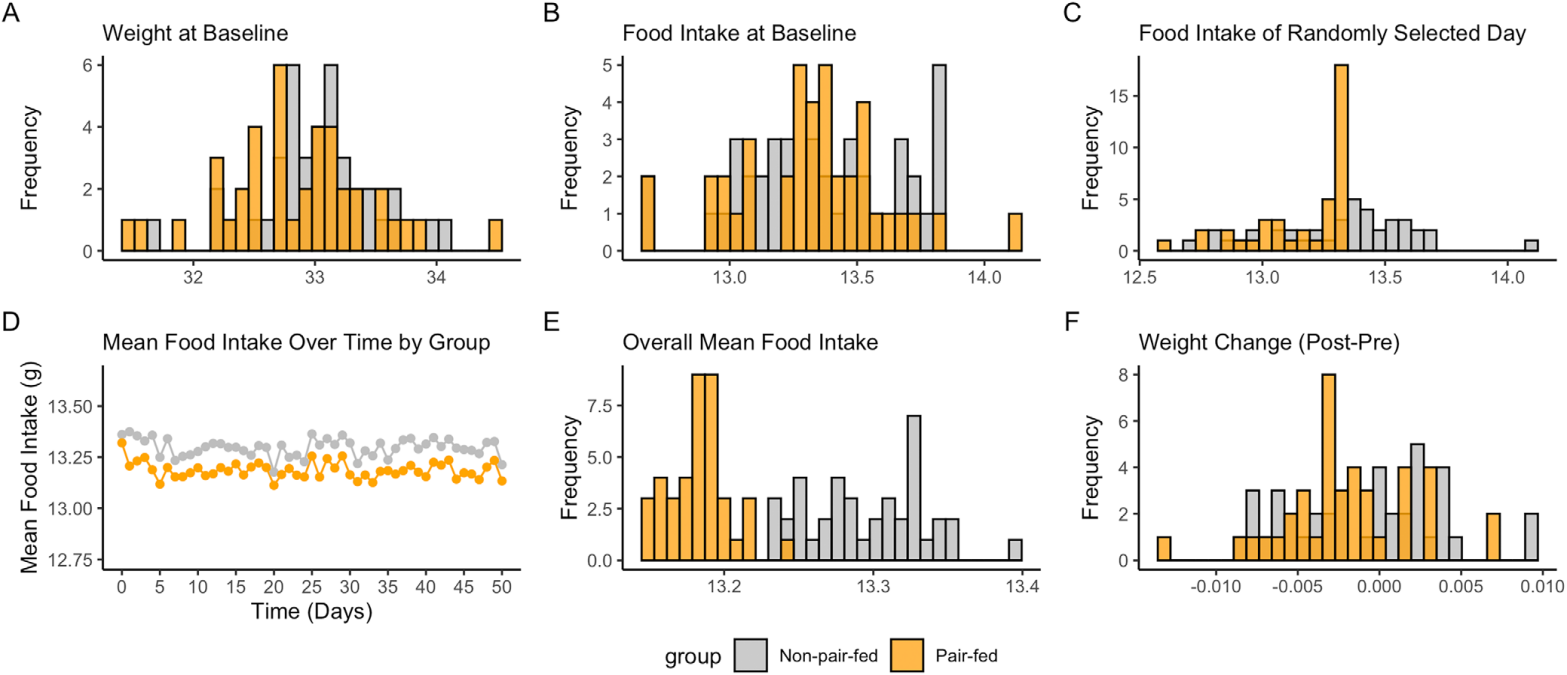
Histograms and line graphs for the distributions of (A) weight at baseline, (B) food intake at baseline, (C) food intake on a randomly selected day, (D) within‐animal mean food intake over time, (E) overall mean food intake, and (F) weight change (post‐pre) in a randomly selected simulated group pair‐feeding dataset. [Color figure can be viewed at wileyonlinelibrary.com]

Figure 1 illustrates the distribution of key variables for the group pair‐feeding scenario. At baseline, the weight (panel A) and food intake (panel B) distributions were similar between the non‐pair‐fed and pair‐fed groups. However, during the study, the pair‐feeding design restricted food intake in the pair‐fed group, leading to a truncated distribution of food intake on a randomly selected day (panel C) and a lower overall mean food intake or within‐animal mean food intake (panel E) compared with the non‐pair‐fed group. Panel D shows that over time the mean food intake of the pair‐fed group was consistently lower than that of the non‐pair‐fed group.

#### 3.1.2 Type I Error Rate

In the group pair‐feeding simulation, analysis of type I error rates revealed significant inflation when food intake was not added as a covariate in the statistical analysis, as shown in Table 1. Across all scenarios, with varying numbers of animals (16 or 80) and days (15 or 50), the type I error rates were significantly inflated in the unadjusted model (ranging from 0.1155 to 0.4251). However, in the model that was adjusted for food intake by adding it as a covariate in the statistical analysis, the observed type I error rates were not statistically significantly different from the nominal rate of 0.05.

**TABLE 1:** Type I error rates in a group pair‐feeding design with varying numbers of animals and days.

Number of animals	Number of days	Type I error rate		*p*_value_1	*p*_value_2
		Model_1	Model_2		
80	50	0.4251	0.0475	< 2.2e−16	0.2513
80	15	0.4192	0.0500	< 2.2e−16	0.9999
16	50	0.1224	0.0517	< 2.2e−16	0.4354
16	15	0.1155	0.0494	< 2.2e−16	0.7831

*Note*: Model_1 is the unadjusted model; Model_2 was adjusted for within‐animal mean food intake. *p*_value_1 and *p*_value_2 assess the statistical significance of the difference between the observed and nominal type I error rate (0.05) for Model_1 and Model_2, respectively.

#### 3.1.3 Individual Pair‐Feeding

**FIGURE 2:**
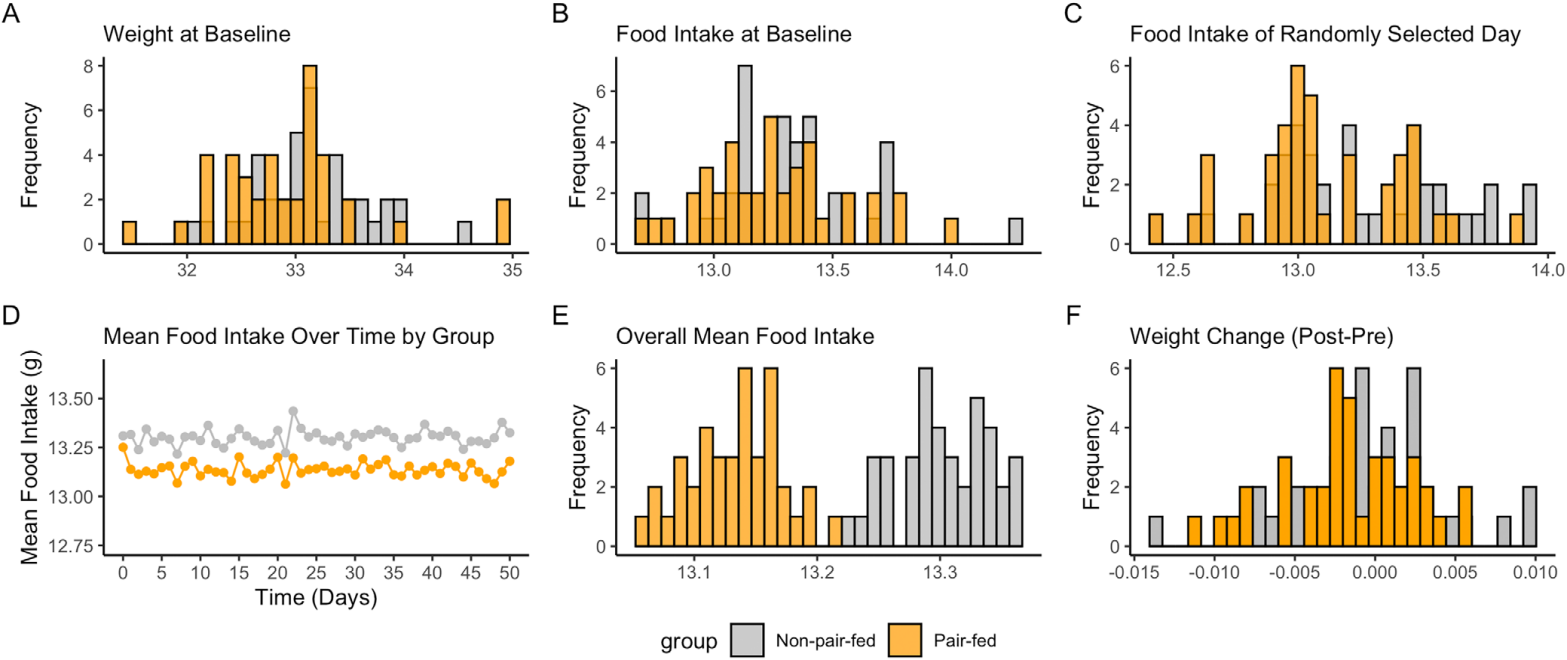
Histograms and line graphs demonstrating the distributions of (A) weight at baseline, (B) food intake at baseline, (C) food intake on a randomly selected day, (D) within‐animal mean food intake over time, (E) overall mean food intake, and (F) weight change (post‐pre) in a randomly selected simulated individual pair‐feeding dataset. [Color figure can be viewed at wileyonlinelibrary.com]

Figure 2 illustrates the distribution of key variables. At baseline, the weight (panel A) and food intake (panel B) distributions were similar between the non‐pair‐fed and pair‐fed groups. During the study, the pair‐feeding design, which paired each animal in the non‐pair‐fed group with an animal in the pair‐fed group, resulted in a truncation of food intake for each animal in the pair‐fed group, as seen in the distribution of food intake from a randomly selected day (panel C). This led to a slightly lower mean food intake over time (panel D) and a different distribution of overall mean food intake or within‐animal mean food intake (panel E) in the pair‐fed group compared with the non‐pair‐fed group.

#### 3.1.4 Type I Error Rate

Table 2 highlights significant inflation in type I error rates for both the crude model (Model_1) and the model adjusted for the pairing variable (Model_2), with error rates ranging from 0.1720 to 0.7137. Adjustment for mean food intake throughout the study (Model_3) reduced the type I error rates with no significant difference from the nominal alpha level of 0.05. However, when the model was adjusted for both mean food intake and the pairing variable (Model_4), the number of animals affected the type I error rate: larger sample sizes (80 animals) showed no significant inflation, whereas smaller sample sizes (16 animals) exhibited significant inflation from the nominal type I error rate.

**TABLE 2:** Type I error rates in an individual pair‐feeding design across varying numbers of animals and days.

Number of animals	Number of days	Type I error rates				*p*_value_1	*p*_value_2	*p*_value_3	*p*_value_4
		Model_1	Model_2	Model_3	Model_4				
80	50	0.6943	0.7137	0.0491	0.0490	< 2.2e−16	< 2.2e−16	0.6796	0.6464
80	15	0.6869	0.7097	0.0484	0.0502	< 2.2e−16	< 2.2e−16	0.4629	0.9269
16	50	0.1779	0.2056	0.0505	0.0588	< 2.2e−16	< 2.2e−16	0.8185	5.397e−05
16	15	0.1720	0.2011	0.0511	0.0626	< 2.2e−16	< 2.2e−16	0.6138	7.414e−09

*Note*: Model_1 is the crude model; Model_2 was adjusted for the pairing variable as a random effect; Model_3 was adjusted for within‐animal mean food intake; Model_4 was adjusted for both within‐animal mean food intake as a covariate and the pairing variable as a random effect. *p*_value_1, *p*_value_2, *p*_value_3, and *p*_value_4 assess the statistical significance of the difference between the observed and nominal type I error rate (0.05) for each model.

#### 3.1.5 Food Intake Differences Between Groups in Pair‐Feeding Designs

**FIGURE 3:**
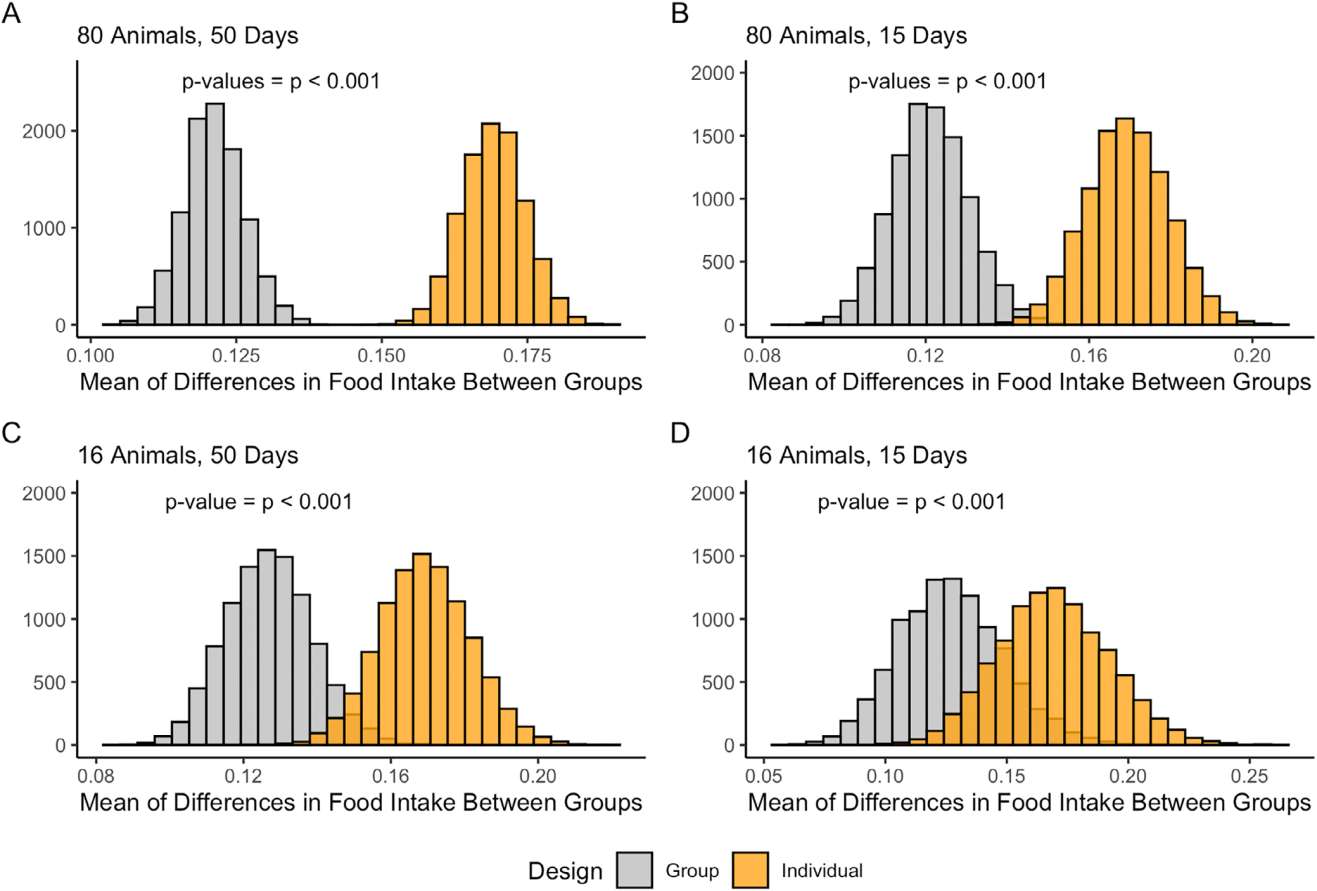
Histograms of the mean of differences in food intake between pair‐fed and non‐pair‐fed groups for individual and group pair‐feeding designs across 10,000 simulations, with *t*‐test results indicating statistical significance of differences between designs. [Color figure can be viewed at wileyonlinelibrary.com]

Figure 3 represents the average of differences in overall mean food intake between the pair‐fed and non‐pair‐fed groups for both group and individual pair‐feeding designs over 10,000 simulations. As the results demonstrate, there was a statistically significant difference between group and individual pair‐feeding designs, with individual pair‐feeding leading to a larger difference in the overall mean food intake between pair‐fed and non‐pair‐fed groups.

### 3.2 Results of Reanalysis of an Existing Experimental Study

**FIGURE 4:**
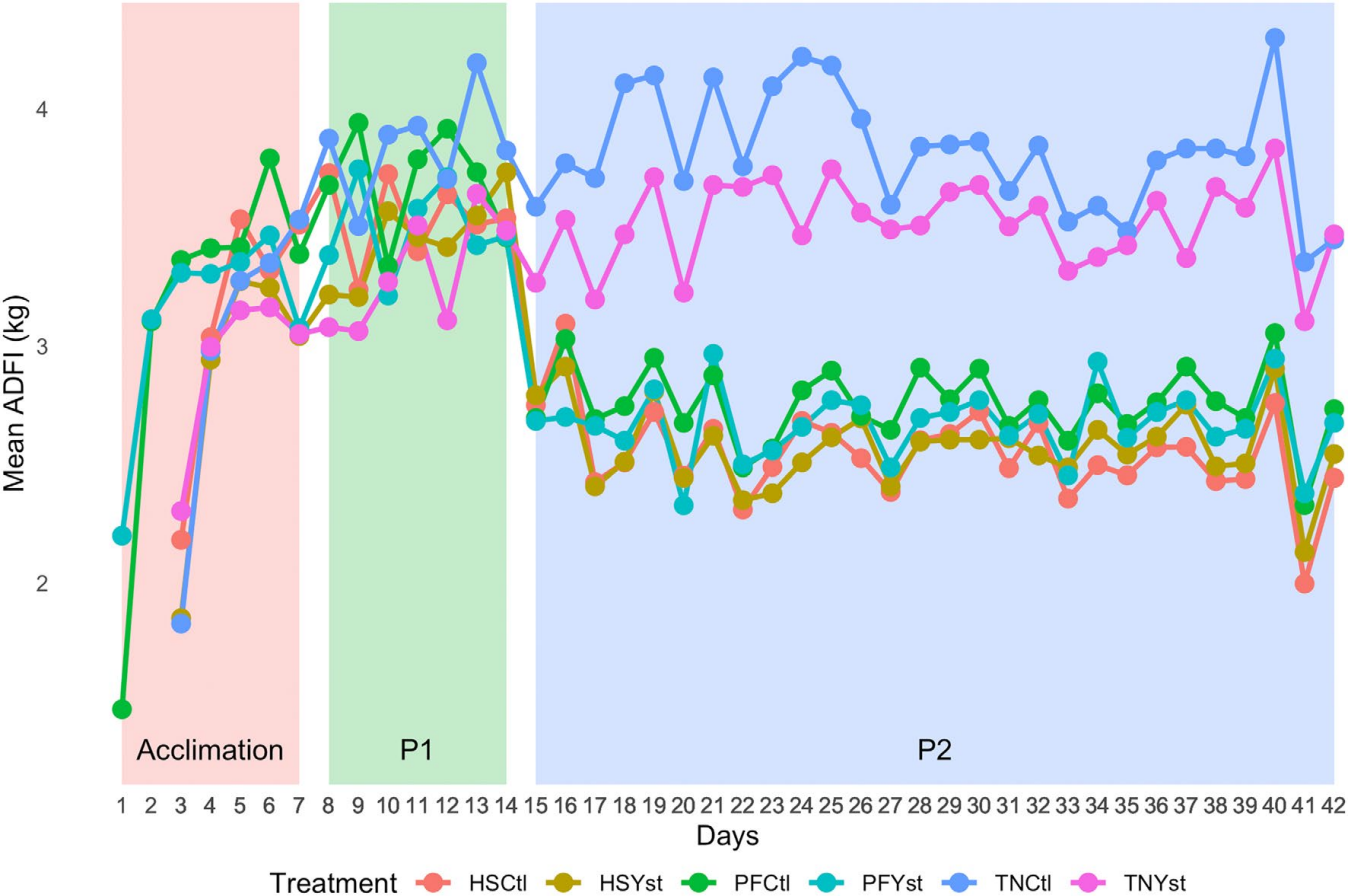
Average daily food intake (ADFI) over time for different treatment groups. HSCtl, heat‐stress control; HSYst, heat‐stress yeast; PFCtl, pair‐fed control; PFYst, pair‐fed yeast; TNCtl, thermoneutral control; TNYst, thermoneutral yeast. [Color figure can be viewed at wileyonlinelibrary.com]

In our reanalysis of data from the group pair‐feeding study by Mayorga et al. [15], we found, as expected, that average daily food intake decreased notably when the intervention started in period 2 among pigs in the heat stress and pair‐fed conditions compared with pigs in the thermoneutral condition. The thermoneutral pigs maintained relatively higher average daily food intake throughout period 2, whereas heat stress and pair‐fed pigs exhibited similar reductions in their food intake. As reported in the original paper, statistical analysis revealed a significant difference in average daily food intake over time of thermoneutral pigs with both heat stress and pair‐fed pigs. However, there was no significant difference in average daily food intake between heat stress and pair‐fed pigs (Figure 4).

Table 3 depicts the differences in average daily gain of body weight across treatment groups for various statistical models. In the published result, the thermoneutral groups (TNCtl and TNYst) showed no significant difference from each other, whereas both differed significantly from the pair‐fed and heat stress groups (PFCtl, PFYst, HSCtl, and HSYst). After we adjusted the model for food intake, however, the results differed from the published results: the difference between the TNCtl and TNYst groups became significant, and their comparison with the PFCtl, PFYst, HSCtl, and HSYst groups also changed.

**TABLE 3:** Reproducibility of the original study's results for average daily body weight gain and reanalysis of the pair‐feeding data.

	Treatment						*p*			Contrasts			
	TNCtl	TNYst	PFCtl	PFYst	HSCtl	HSYst	Trt	Week	Trt × Week	TN vs. PF	TN vs. HS	PF vs. HS	Ctl vs. Yst
Published	1.04	1.08	0.71	0.69	0.77	0.79	< 0.001	< 0.001	< 0.001	< 0.001	< 0.001	0.02	0.163
Pig as random effect	1.04	1.08	0.71	0.69	0.77	0.79	< 0.001	< 0.001	< 0.001	< 0.001	< 0.001	0.03	0.67
Adjustment for food intake	0.80	0.90	0.75	0.75	0.85	0.86	0.001	< 0.001	< 0.001	0.02	0.97	0.0005	0.085
Pig as random effect and adjustment for food intake	0.79	0.90	0.75	0.75	0.85	0.86	0.007	< 0.0001	< 0.001	0.048	0.80	0.002	0.11

*p* for between group comparisons				
	Published	Pig as random effect	Adjustment for food intake	Adjustment for pig as random effect and food intake
TNCtl vs. TNYst	0.5153	0.5153	0.0166	0.0275
TNCtl vs. PFCtl	< 0.0001	< 0.0001	0.3151	0.4568
TNCtl vs. PFYst	< 0.0001	< 0.0001	0.3503	0.5016
TNCtl vs. HSCtl	< 0.0001	< 0.0001	0.3745	0.3101
TNCtl vs. HSYst	< 0.0001	< 0.0001	0.2864	0.2416
TNYst vs. PFCtl	< 0.0001	< 0.0001	0.0016	0.0065
TNYst vs. PFYst	< 0.0001	< 0.0001	0.0025	0.0093
TNYst vs. HSCtl	< 0.0001	< 0.0001	0.2685	0.4005
TNYst vs. HSYst	< 0.0001	< 0.0001	0.3431	0.4823
PFCtl vs. PFYst	0.7262	0.7262	0.9518	0.9422
PFCtl vs. HSCtl	0.2018	0.2018	0.0085	0.0170
PFCtl vs. HSYst	0.1034	0.1034	0.0049	0.0112
PFYst vs. HSCtl	0.1018	0.1018	0.0097	0.0199
PFYst vs. HSYst	0.0477	0.0477	0.0057	0.0134
HSCtl vs. HSYst	0.6125	0.6125	0.7682	0.8047

*Note*: TNCtl, thermoneutral control; TNYst, thermoneutral yeast; PFCtl, pair‐fed control; PFYst, pair‐fed yeast; HSCtl, heat‐stress control; HSYst, heat‐stress yeast. Trt, Treatment. TN, TNCtl + TNYst; PF, PFCtl + PFYst; HS, HSCtl + HSYst; Ctl, TNCtl + PFCtl + HSCtl; Yst, TNYst + PFYst + HSYst.

## 4 Discussion

Our study has shown that group and individual pair‐feeding designs are susceptible to inflated T1Er rates under some circumstances when food intake is not accounted for in the statistical analysis. In the group pair‐feeding scenario, T1Er rates from the unadjusted model ranged from 0.1155 to 0.4251 with various combinations of sample sizes and durations, which differed significantly from the nominal T1Er rate of 0.05. Adjusting for food intake corrected this inflation, resulting in no significant differences from nominal T1Er rates. A similar pattern was observed in the individual pair‐feeding design. Controlling for food intake with or without adjusting for pairing corrected the T1Er inflation in simulations with large sample sizes regardless of study duration. However, with small sample sizes, adjusting for pairing alone or for both pairing and food intake did not correct the inflation, whereas adjusting for food intake alone reduced the T1Er inflation to nominal T1Er rates.

Our reanalysis of a randomized experimental group pair‐feeding study illustrates that failure to properly account for food intake can affect the conclusions drawn about treatment effects. In the original study analysis, most of the differences of the TNYst and TNCtl groups with all other groups were significant and were due to the differences in food intake. In our analyses, when we adjusted for food intake, these significant differences of TNYst and TNCtl with all other groups became statistically insignificant. However, the differences between the pair‐fed and non‐pair‐fed groups (PFCtl vs. HSCtl and PFYst vs. HSYst) became significant after we appropriately adjusted for food intake, suggesting strong evidence against the null hypothesis.

Our findings illustrate that differences in food intake distributions can exist between pair‐fed and non‐pair‐fed groups and may even be induced by the pair feeding itself. If such differences are not accounted for statistically, they can introduce bias when estimating treatment effects. Therefore, testing the null hypothesis of no effect of treatment assignment on the outcome of interest can inflate type I error rates. Broadly speaking, this inflation can be attributed to violations of the “no interference” component of SUTVA, because the feeding behavior of one group or subject directly interferes with the food intake of another group or subject. Our results suggest caution against this assumption and show that if food intake is not explicitly and appropriately accounted for, the intervention's effect can be misestimated. Therefore, the appropriate null hypothesis to be tested when conducting a pair‐feeding study is no effect of treatment assignment on the outcome of interest *independent of observed food intake*.

It is important to note that our study does not claim that pair feeding designs will always create differences in food intake between pair‐fed and non‐pair‐fed groups; rather, it illustrates that such differences can and often do occur. Not accounting for these differences statistically can bias the treatment effect estimate. If food intake were truly identical between groups in pair‐feeding designs, then no bias would result from food intake. Moreover, pair feeding not only changes the metabolizable energy available, but also the manner in which food is delivered, which itself may influence outcomes. For instance, the perception of having greater or limited access to food can affect physiology beyond actual intake, as shown in prior studies [19]. Thus, pair feeding designs could introduce biases not just statistically, but also by influencing the physiologic state of the animal.

Although some may argue that if pair feeding results in equal body weights between groups, then adjustment for food intake is unnecessary, this is not necessarily the case. First, body weight and food intake are distinct, and although they are typically correlated, the relationship is not one‐to‐one. Some investigators may be interested in the effect of treatment on distal outcomes independent of food intake; others, in the effect of treatment on distal outcomes independent of body weight. What needs to be controlled for depends on the research question. Second, animals having equal body weights on average do not mean that food intake has been controlled for (or even body weight itself). Body weight and food intake are time‐varying covariates. Equivalence in mean values at one or more time points does not guarantee equivalence at every time point. Looking for approximate equivalence across groups is not the same as statistical control. Statistical control could, in principle, incorporate multiple measurements and functions of those measurements across time, which need not be simple linear averages. Third, equality of means is not the same as equality of distributions. Nonlinear effects will be missed if one compares only group means. Methods such as transformations, polynomial terms, quantile regression, or general lambda distribution regression allow a richer modeling of such cases. Fourth, it is important to distinguish between exact equality and the absence of statistically significant differences. In practice, it is unlikely that two sample means will ever be exactly equal. Relying only on statistical significance to answer “how equal is enough?” is not appropriate, as has been widely noted in the literature. Fifth, equality in an observed sample is not the same as equality in probability across the distribution of all possible randomizations and study replications. It is this latter concept of equality in probability that underlies inference and motivates the use of statistical control. Finally, equality (or lack thereof) of variables does not necessarily imply bias (or lack thereof). For all these reasons, we believe it is meaningful to consider adjustment for food intake even when body weights are approximately equal in a pair‐fed design.

Several limitations should be considered when interpreting these findings. First, our simulations were based on simplified assumptions regarding the distribution of food intake and its relationship with the outcome. In real experimental contexts, food intake may be influenced by behavioral, metabolic, and/or environmental factors, and food intake may have complex relationships with the outcome of interest that are difficult to capture in a simulation. To paraphrase Mook [20], we have shown what can happen, not necessarily what will happen. Additionally, in some pair‐feeding studies, animals may be housed in groups rather than individually [21]. Group housing may induce correlations or dependencies among animals, which further affect the validity of statistical conclusions with pair feeding.

We focused on T1Er rates. Yet, pair‐feeding might also affect type II error rates, confidence interval coverage, standard error estimates, and parameter point estimates. Such effects merit evaluation in future research. Moreover, we have described the results we obtained under the broad umbrella of SUTVA violations. Whether adjustment for food intake truly resolves the SUTVA (no‐interference) violation is questionable. There are at least three reasons adjustment may be incomplete. First, proper adjustment requires modeling food intake with the correct functional form. If the true relationship between food intake and the outcome is not linear, but we adjust only linearly, then the adjustment would be incomplete. Second, if food intake is measured with error, which it almost certainly is, we will not have fully adjusted for it. Although such error can, in principle, be modeled, doing so requires more complex models and knowledge of the measurement error. In rodent studies, these errors are likely small. Third, interference may influence not only expected values or distributions but also the independence of residuals, thereby potentially violating the assumption of independent observations. Additional deviations in statistical conclusion validity that may exist include: (a) altering the shape or variance of the food intake distribution in the pair‐fed group; (b) creating heterogeneity between the pair‐fed and non‐pair‐fed groups with respect to the shape or variance of the food intake distribution; and (c) reducing the residual variance in the outcome (e.g., weight), which can increase T1Er under a biased null and reduce the type II error rate (i.e., increase power) under the alternative hypothesis (cf., Yu et al. [22]).

Finally, one implication of this study is to use pair feeding in a way that leads to almost no differences in food intake between groups. One example would be to provide low enough amounts of food so that all animals, regardless of treatment group, consume virtually all the food with minimal spillage. Another implication is to not use pair feeding but rather measure food intake and then statistically adjust for it. Whether combining pair feeding with statistical control for food intake provides advantages over statistical control alone is an interesting question that we recommend as a topic for future research.

## 5 Conclusion

Our study demonstrated that pair‐feeding, while used to eliminate biases due to food intake differences across groups, can under some circumstances actually induce such biases and inflate T1Er rates. This is especially the case if individual food intake is not measured and appropriately accounted for in the statistical analysis.

## Author Contributions

Conceptualization: D.B.A. and W.N. Methodology: W.N., D.B.A., B.R., D.E.K., L.G.‐A., D.K.T., L.W.‐T.F., and J.G.I. Formal analysis: W.N., D.E.K., and L.G.‐A. Software: W.N. Validation: W.N. Data curation: W.N., L.H.B., and E.J.M. Writing – original draft preparation: W.N. Writing – review and editing: D.B.A., L.W.‐T.F., J.G.I., B.R., D.E.K., D.K.T., L.G.‐A., L.H.B., and E.J.M. Visualization: W.N. Supervision: D.B.A. Project administration: D.B.A. and W.N. Funding acquisition: D.B.A. All authors have read and agreed to the published version of the manuscript.

## Conflicts of Interest

D.B.A. reports having received funding or payments from numerous government, nonprofit, and for‐profit organizations, but none of which relates directly to the topic herein. The other authors declare no conflicts of interest.

## Supporting information


**Appendix S1:** oby70079‐sup‐0001‐AppendixS1.docx.

## Data Availability

Data sharing not applicable to this article as no datasets were generated or analyzed during the current study.
