# Supplementary material for: Pair‐Feeding Study Designs Can Create Biases and Inflate Type I Error Rates: A Simulation Study
Source: Obesity (Silver Spring). 2025 Nov 24;34(2):407–16. doi: 10.1002/oby.70079 (PMC12850568; doi:10.1002/oby.70079)
Supplement: Supplementary file 1 — Appendix S1: oby70079‐sup‐0001‐AppendixS1.docx. [file OBY-34-407-s001.docx]

**Appendix**

**Statistical Models Used for Evaluating Weight Change**

This section provides the exact statistical models used to estimate the treatment effect on body weight change (ΔW) under both the group and individual pair-feeding designs. In all models, the treatment assignment effect is represented by *β*_1_​, and type I error rate evaluations are based on this coefficient.

### **Notation Summary**

- $\Delta W_{j}:$ Weight change for animal$j$
- $X_{j}:$ Treatment indicator (0 = control, 1 = treatment)
- $\bar{F}_{j}:$ Mean food intake of animal $j$
- $u_{p}:Random intercept for pair p$
- $\epsilon_{j}:residual errors$

1. **Group Pair-Feeding Design**: Two linear regression models were used:
   1. Model 1.1: Linear Regression without Covariates

$$\Delta W_{j}: \beta_{0}+\beta_{1}X_{j}+\epsilon_{j}$$

- This model estimates the treatment effect without adjusting for any additional variables.
  1. Model 1.2: Linear Regression with Mean Food Intake as Covariate

$$\Delta W_{j}: \beta_{0}+\beta_{1}X_{j}+\beta_{2}\bar{F}_{j}+\epsilon_{j}$$

- This model estimates the treatment effect with adjusting for mean food intake.

1. **Individual Pair-Feeding Design**: Four regression models were used:
2. Model 2.1: Linear Regression without Covariates.

$$\Delta W_{j}: \beta_{0}+\beta_{1}X_{j}+\epsilon_{j}$$

- This model estimates the treatment effect without adjusting for any additional variables.

1. Model 2.2: Linear Regression with Mean Food Intake as Covariate.

$$\Delta W_{j}: \beta_{0}+\beta_{1}X_{j}+\beta_{2}\bar{F}_{j}+\epsilon_{j}$$

- This model estimates the treatment effect with adjusting for mean food intake.

1. Model 2.3: Linear Mixed-Effects Model with Random Pair Effect.

$$\Delta W_{jp}: \beta_{0}+\beta_{1}X_{jp}+u_{p}+\epsilon_{jp}$$

- This model estimates the treatment effect with adjusting for pairing as random effect.

1. Model 2.4: Linear Mixed-Effects Model with Random Pair Effect and Mean Food Intake as Covariate.

$$\Delta W_{jp}: \beta_{0}+\beta_{1}X_{jp}+\beta_{2}\bar{F}_{jp}+u_{p}+\epsilon_{jp}$$

- This model estimates the treatment effect with adjusting for mean food intake as fixed effect and pairing as random effect.
